# Cell surface marker profiling of human tracheal basal cells reveals distinct subpopulations, identifies MST1/MSP as a mitogenic signal, and identifies new biomarkers for lung squamous cell carcinomas

**DOI:** 10.1186/s12931-014-0160-8

**Published:** 2014-12-31

**Authors:** Emily Van de Laar, Monica Clifford, Stefan Hasenoeder, Bo Ram Kim, Dennis Wang, Sharon Lee, Josh Paterson, Nancy M Vu, Thomas K Waddell, Shaf Keshavjee, Ming-Sound Tsao, Laurie Ailles, Nadeem Moghal

**Affiliations:** Department of Medical Biophysics, Ontario Cancer Institute/Campbell Family Cancer Research Institute/Princess Margaret Cancer Centre/University Health Network, University of Toronto, Toronto, ON M5G 1 L7 Canada; Department of Applied Mathematics, University of Waterloo, 200 University Avenue West, Waterloo, ON N2L 3G1 Canada; Department of Oncological Sciences, Huntsman Cancer Institute, University of Utah, Salt Lake City, UT 84112 USA; Toronto Lung Transplant Program, University Health Network, University of Toronto, Toronto, ON M5G 1 L7 Canada; Present address: Helmholtz Zentrum München, Institute of Stem Cell Research, Ingolstädter Landstrasse 1, 85746 Neuherberg, Germany; Present address: University of Utah School of Medicine, Salt Lake City, UT 84132 USA; Present address: Ontario Cancer Institute and Princess Margaret Hospital, University Health Network, Toronto, ON M5G 1 L7 Canada

## Abstract

**Background:**

The large airways of the lungs (trachea and bronchi) are lined with a pseudostratified mucociliary epithelium, which is maintained by stem cells/progenitors within the basal cell compartment. Alterations in basal cell behavior can contribute to large airway diseases including squamous cell carcinomas (SQCCs). Basal cells have traditionally been thought of as a uniform population defined by basolateral position, cuboidal cell shape, and expression of pan-basal cell lineage markers like KRT5 and TP63. While some evidence suggests that basal cells are not all functionally equivalent, few heterogeneously expressed markers have been identified to purify and study subpopulations. In addition, few signaling pathways have been identified that regulate their cell behavior. The goals of this work were to investigate tracheal basal cell diversity and to identify new signaling pathways that regulate basal cell behavior.

**Methods:**

We used flow cytometry (FACS) to profile cell surface marker expression at a single cell level in primary human tracheal basal cell cultures that maintain stem cell/progenitor activity. FACS results were validated with tissue staining, *in silico* comparisons with normal basal cell and lung cancer datasets, and an *in vitro* proliferation assay.

**Results:**

We identified 105 surface markers, with 47 markers identifying potential subpopulations. These subpopulations generally fell into more (~ > 13%) or less abundant (~ < 6%) groups. Microarray gene expression profiling supported the heterogeneous expression of these markers in the total population, and immunostaining of large airway tissue suggested that some of these markers are relevant *in vivo*. 24 markers were enriched in lung SQCCs relative to adenocarcinomas, with four markers having prognostic significance in SQCCs. We also identified 33 signaling receptors, including the MST1R/RON growth factor receptor, whose ligand MST1/MSP was mitogenic for basal cells.

**Conclusion:**

This work provides the largest description to date of molecular diversity among human large airway basal cells. Furthermore, these markers can be used to further study basal cell function in repair and disease, and may aid in the classification and study of SQCCs.

**Electronic supplementary material:**

The online version of this article (doi:10.1186/s12931-014-0160-8) contains supplementary material, which is available to authorized users.

## Background

The primary function of the lungs is to deliver oxygen to blood and remove carbon dioxide. This occurs by the conduction of air from the larger airways (e.g. trachea, bronchi, and bronchioles) to the smaller alveoli where gas exchange occurs with the pulmonary capillaries. To perform this function, the airways are lined with distinct specialized epithelia in different anatomical segments. The nasopharyngeal, tracheal, and bronchial epithelia (tracheobronchial epithelia) are similar and have a cellular composition that prevents infection from inhaled particles and microorganisms. Secretory cell types within this epithelium produce an air surface liquid, which traps microorganisms and is expelled by the coordinated beating of cilia on ciliated cells. Abnormalities in the regeneration of this epithelium contribute to the pathogenesis of large airway diseases such as COPD, asthma, cystic fibrosis, and squamous cell carcinomas.

The tracheobronchial epithelium is derived from basal cells, which are marked by CD44, KRT5, and the TP63 transcription factor [1-4]. In mice, lineage tracing of KRT5-expressing cells indicates these cells self-renew and give rise to secretory and ciliated cells [5]. These findings are also supported by KRT14 lineage tracing, which marks a subset of basal cells [6,7]. Similarly, when analyzed *in vivo* in denuded rat tracheas, human tracheal basal cells generate secretory and ciliated cells, but also give to rise to submucosal glands beneath the surface epithelium [8]. In addition, *in vitro*, clonally expanded TP63-positive human tracheobronchial basal cells differentiate into secretory and ciliated cells when grown under air-liquid-interface culture conditions [9]. Whether all TP63-positive basal cells have equivalent stem/progenitor activity and what are the signals that regulate their self-renewal and differentiation are major questions in large airway stem cell biology. In rat tracheal xenografts, individual human tracheobronchial basal cells generate different numbers of progeny with distinct combinations of basal, ciliated, goblet, and submucosal gland cells, suggesting heterogeneity in function [8]. Some of these differences may track with differences in aldehyde dehydrogenase activity, which varies among basal cells and correlates with better growth *in vitro* [10]. Similarly, murine tracheal basal cells may also be functionally heterogeneous, with some KRT5-expressing cells growing better than others *in vitro* [5]. In this setting, KRT14 appears to be another marker of heterogeneity since KRT5/KRT14-double positive cells are a minor population in the normal epithelium, but become abundant after injury [11-13].

Whether functionally distinct responses of individual basal cells arise stochastically, hierarchically, or from environmentally-regulated differences in cell state is not known. There has been little phenotypic characterization of basal cell diversity, and distinct subsets of molecularly-defined basal cells have generally not been purified to homogeneity. Studies of basal cell diversity have been hampered by difficulties with *in vivo* studies, potential species-specific differences in basal cell properties, and a lack of tools. Certain basal cell subpopulations might be rare under normal uninjured conditions, and in general, tissue antibody staining can be problematic. Furthermore, the compositions of the tracheobronchial epithelia are not identical between humans and all animals. As compared to mice, human large airways have more goblet cells, and basal cells and submucosal glands extend more distally; while murine large airways have abundant Clara cells, a secretory cell type that is confined to bronchioles in humans [14-17]. These observations suggest that basal cells might have species-specific differences in lineage potential and/or regulation, which is supported by comparisons of their transcriptomes [18]. Indeed, there appears to be differences in expression of keratins, integrins, developmental transcription factors (e.g. FOX family, SOX family, HMG family) and signaling molecules (e.g. Wnt family) between human and murine basal cells.

To date, only a small number of soluble factors have been identified that regulate human tracheobronchial basal cell proliferation and differentiation. EGF, insulin, and FGF7 are mitogens for basal cells [19-22], while TFF3 stimulates ciliogenesis and IL-4 and IL-13 promote mucinous differentiation [23-25]. Retinoic acid (RA) promotes differentiation both of ciliated and mucinous cell types [26,27]. Here, we performed the first large scale profiling of cell surface marker expression at a single cell level in primary cultures of human tracheal basal cells. We found 105 markers that were expressed on all tested isolates of basal cells, with 47 markers partitioning basal cells into subpopulations of different sizes. The expression of some markers was confirmed on basal cells *in vivo*. Furthermore, 24 markers were enriched in lung squamous cell carcinomas (SQCCs) relative to adenocarcinomas, and four markers had significant associations with overall survival for SQCC patients. Finally, we found that the MST1R/RON receptor tyrosine kinase is expressed on all basal cells, and identified its ligand MST1/MSP, as a new mitogen for tracheobronchial basal cells. These cell surface markers will now allow the purification and study of molecularly-defined subsets of human basal cells, identify new signaling pathways that may regulate proliferation and differentiation, and may also aid in the classification and study of SQCCs.

## Methods

### Ethics statement and human tracheal tissue

Normal human tracheal tissue was obtained as surgical waste from lung transplant operations with written informed consent from lung donors, and with the approval of the University Health Network Research Ethics Board (study 08-0318-T). The tracheal tissue was healthy and was obtained from lungs deemed to be of suitable quality for lung transplants, but was not necessary for the transplant procedure. For high throughput antibody screening, the three strains of basal cells were derived from patients with the following characteristics: Strain 23 (age 47, male, non-smoker), Strain 38 (age 59, male, non-smoker), and Strain 37 (age 38, male, non-smoker). The 8227 leukemic cell line was derived from a primary human AML with written informed consent from the donor, and with approval of the University Health Network Research Ethics Board (study 02-0763-C).

### Isolation of human tracheal epithelial cells

Basal cells were isolated following the procedure of Karp et al. [28]. Briefly, tracheal tissue was digested with 1.4 mg/ml Protease type XIV (Sigma) in minimal essential media at 4°C for 24–48 hours. After digestion, tracheal epithelial cells were pelleted, rinsed with minimal essential medium, resuspended in LHC-9 media [20] with 10% fetal bovine serum and 10% dimethylsulfoxide, and stored in cryovials in a liquid nitrogen freezer.

### Tracheal basal cell culture

Primary tracheal basal cells were expanded in LHC-9 media containing 50 μg/ml gentamycin, 1 unit/ml penicillin, 1 μg/ml streptomycin, and 1.25 μg/ml amphotericin B, and additional supplements as described [20], on plastic Petri dishes coated with 48 μg/ml PureCol (Advanced BioMatrix) diluted in 0.01 N hydrochloric acid. Cells were grown at 37°C in a 5% CO_2_ incubator, and media was changed every other day. To subculture basal cells, cells were treated with 0.025% trypsin/EDTA, which was subsequently neutralized with an equal volume of Trypsin Neutralizing Solution (Lonza).

### Xenografts

Rat tracheal xenografts were performed as described [29,30] with the approval of the UHN Animal Care Committee. Briefly, the endogenous epithelium from adult rat tracheas was removed by three rounds of freeze/thaw. 1 × 10^6^ primary human tracheal basal cells were seeded per trachea, and one tracheal cassette was implanted subcutaneously onto the back of a single adult male NOD/SCID mouse. After implantation, tracheas were irrigated once per week with Ham’s F-12 media, and after three weeks, were irrigated twice per week. Tracheas were harvested between five to eight weeks after transplantation.

### Immunofluorescence, histology, and immunohistochemistry

For immunofluorescence staining of primary tracheal basal cell cultures, basal cells were cytospun onto charged glass slides and fixed in 4% paraformaldehyde for 10 minutes at room temperature. Following fixation, excess paraformaldehyde was quenched by three five minute washes in 100 mM glycine/PBS. Cells were permeabilized with 0.1% Triton X-100/PBS, blocked with 3% bovine serum albumin (BSA)/0.1% Triton X-100/PBS for 30 minutes at room temperature, and then incubated with primary antibody diluted in 3% BSA/0.1% Triton X-100/PBS for one hour at room temperature. Primary antibodies were α-KRT5 (ab24647, Abcam, 1:100), α-TP63 (sc-56188, Santa Cruz, 1:100), α-MUC5AC (MS-145-P1, Neomarkers, 1:100), α-MUC16 (sc-33344, Santa Cruz, 1:100), and α-BTUB4 (T7941, Sigma, 1:100). After primary antibody incubation, cells were washed three times with 0.1% Triton X-100/PBS and incubated with secondary antibody in 3% BSA/0.1% Triton X-100/PBS for two hours at room temperature or overnight at 4°C. Secondary antibodies were Alexafluor 488 goat anti-rabbit (Invitrogen, 1:500) and Alexafluor 568 goat anti-mouse (Invitrogen, 1:500). After secondary antibody staining, cells were washed three times with 0.1% Triton X-100/PBS and mounted in Vectashield mounting media containing DAPI (Vector Laboratories).

Human tracheal and rat tracheal xenograft tissue were fixed in 10% buffered formalin overnight at room temperature, then soaked in 70% ethanol overnight at room temperature, and then paraffin embedded. Paraffin blocks were cut into 8 μm sections, placed on positively charged glass slides, and then dried at 55°C overnight. For immunofluorescence staining, sections were deparaffinized through successive incubations in xylene and decreasing concentrations of ethanol and antigens were retrieved in 10 mM citrate buffer, pH 6.0, using the 2100 Retriever (Aptum Biologics, Ltd.). Hematoxylin/eosin staining was performed using standard methods. Anti-MST1R/RON (Santa Cruz, sc-322, 1:400) immunohistochemistry was performed using the Vantana Benchmark XT autostainer with the iVIEW DAB detection kit (Vantana Medical Systems). Images were captured using an Axioimager Z1 microscope and either an AxioCam MRm camera with Axiovision software (Zeiss) or a Canon EOS color camera.

Fluorescence In Situ Hybridization (FISH) to detect human DNA in xenografted rat tracheal epithelia was performed on paraffin-embedded tissue using the human specific CEP3 Alpha Satellite DNA Spectrum Orange probe (Abbott Molecular) and the manufacturer’s instructions.

### High-throughput antibody screen and FACS

Primary human tracheal basal cells were expanded on plastic until ~70% confluent (7–10 days), removed with 0.025% trypsin, incubated with trypsin neutralizing solution, and then pelleted and resuspended in FC buffer (Hanks buffered salt solution with 1% fetal bovine serum). In control experiments with antibodies that detected heterogeneously expressed markers, we found that of 64 tested antibodies, only three (4.7%) detected epitopes that were trypsin-sensitive (Additional file 1: Table S3). Furthermore, this sensitivity was seen with a two-fold higher concentration of trypsin (0.05%), and was only partial, with marker expression reduced 2-3-fold. Thus, our trypsinization conditions did not have a major effect on marker detection. High-throughput staining and analysis were generally performed as described [31]. 50,000 tracheal basal cells in 100 μl were aliquoted per well into round-bottom 96-well plates, with each well containing a 2X concentration of each of 334 different fluorochrome-conjugated cell-surface targeted antibodies (PE, FITC, or APC; see Additional file 2: Table S1 for vendors). Wells containing buffer only were included as negative controls. The final antibody dilution was 1:50 for all antibodies. Staining was performed on ice for 20 minutes, after which plates were centrifuged, buffer aspirated, and pellets washed twice with 200 μl of FC buffer. Finally, pellets were resuspended in 100 μl of FC buffer containing 1 μg/ml of DAPI to allow exclusion of dead cells. Cells were then analyzed on a BD LSRII equipped with a high throughput sampler. A minimum of 10,000 events per well were collected using FACSDiva software, and resulting FCS 3.0 files exported to FlowJo version 9.3 for analysis. Debris, dead cells (DAPI-positive), and live cell doublets were excluded from analysis by using the gating strategy outlined in Additional file 3: Figure S1. Validation experiments were either performed in a high-throughput manner (with Strain 37) or a single tube format. For single tube staining, approximately 2 × 10^5^ cells were stained in 100 μl of FC buffer with a 1:50 dilution of the test antibody. Alternatively, α-CD45-APC (555485, BD Pharmingen) and α-CD326/EpCAM-FITC (sc-66020, Santa Cruz) were used at 1:10 and 1:25, respectively. CD44 was detected with α-CD44 (14-0441-81, eBioscience, 1:50) and goat α-mouse-APC (BD Pharmingen, 1:500).

### Human protein atlas images

Images were downloaded from the online resource at www.proteinatlas.org. Antibodies used in the downloaded images included EGFR (Zymed, 28–0005), MST1R/RON/CD136 (SDIX, 1659.00.02), PDPN (MBL, D189-1), CD6 (Santa Cruz, sc-65249), CD9 (Novocastra, NCL-CD9), CD49C (Sigma, HPA008572), CD51 (Novocastra, NCL-CD51), CD54 (Sigma, HPA004877), CD55 (Sigma, HPA024386), CD66C (Sigma, HPA011041), CD71 (Zymed, 13–6800), CD91 (Sigma, HPA022903), CD99 (Dako Cytomation, M3601), CD116 (Santa Cruz, sc-21764), CD117 (Dako/Cytomation, A4502), CD138 (Sigma, HPA006185), CD142 (Santa Cruz, sc-20160), CD146 (Novocastra, NCL-CD146), CD226 (Sigma, HPA015715), CD227 (Dako Cytomation, M0613), CD268 (MBL, D201-3), CD276 (Sigma, HPA009285).

**Figure 1:**
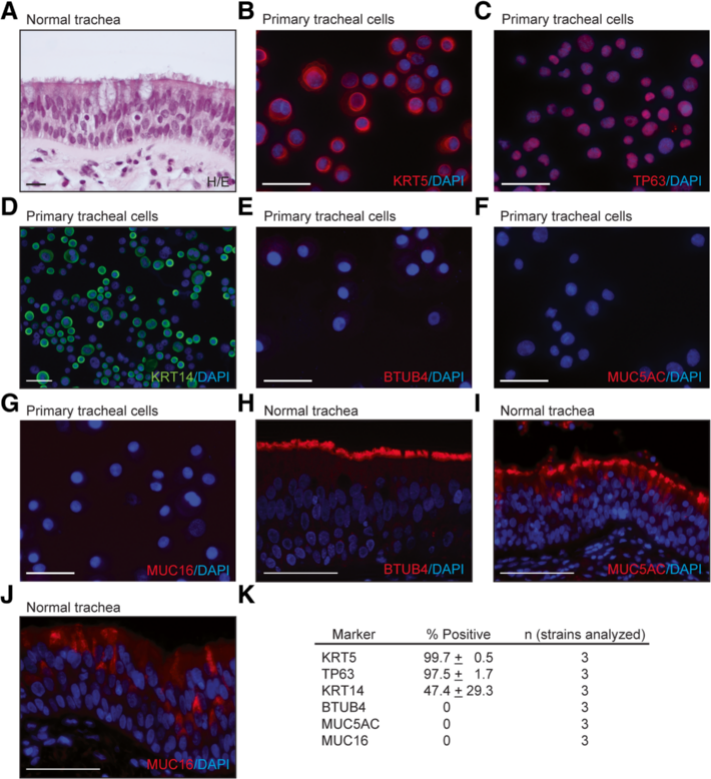
**Primary human tracheal epithelial cell cultures are pure populations of basal cells.** Primary human tracheal epithelial cells growing on plastic were cytospun onto glass slides and stained with the indicated antibodies **(B-G)**. Staining of control human tracheal tissue **(A, H-J)**. **(K)** Quantification of lineage marker expression in human tracheal basal cell isolates derived from different donor tracheas. For each strain, 300–800 cells were scored. Mean frequencies ± standard deviations between the strains are shown. Scale bars are 50 μm.

**Figure 2:**
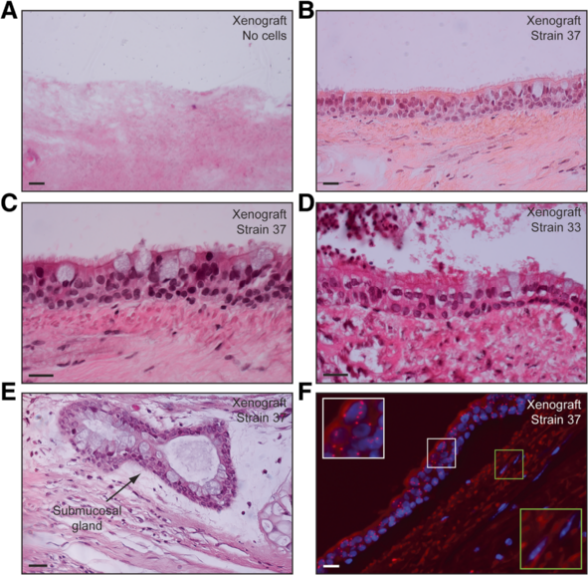
**Conditions that support expansion of primary human tracheal basal cells maintain mucociliary differentiation activity.** Expanded, primary P0 human tracheal basal cells were enzymatically removed from plastic dishes and grown as rat tracheal xenografts. After five weeks, mucociliary differentiation in xenografts was assessed morphologically by H/E staining of tissue sections **(A, B, D-F)**. **(A)** Control transplanted rat tracheal xenograft not seeded with human basal cells. **(C)** FISH with a human-specific chromosome 3 centromeric probe to detect cells of human origin. Insets show magnified areas of positivity in xenografted epithelia (white), but not underlying stroma (green). Scale bars are 20 μm.

### Gene expression analysis

To examine surface marker gene expression in cultured, normal human bronchial basal cells, microarray gene expression data were downloaded from GEO (http://www.ncbi.nlm.nih.gov/geo/) (accession number: GSE24337) [18]. All of the surface markers that were expressed in three or more isolates of basal cells (Additional file 6: Table S2), as well as all of the markers representing receptors for cleaved or secreted factors (Table 1), were selected for mRNA expression analysis. Surface marker genes were mapped to Affymetrix probes using ArrayTrans (http://www.ocigc.ca/arraytrans/). 111/119 of these markers could be mapped to microarray probes. mRNA expression values were log normalized, and for each gene, the probe data with the largest expression value was used. A Pearson’s product moment correlation coefficient was then used to test for association between mRNA expression for a given marker and its average frequency in the population of normal basal cells (as determined by FACS).

To examine surface marker gene expression in primary lung cancers, RNAseq data (Tier 3) for adenocarcinomas and squamous cell carcinomas from The Cancer Genome Atlas were downloaded from cBioportal (http://www.cbioportal.org) [32]. For this analysis, 110 out of the above 119 markers could be mapped to genes in the RNAseq data. Log quantile normalized RSEM values were used for testing differential expression between the two cell types, and a Bonferroni correction was used to adjust p-values for multiple testing.

### Validation of alamarBlue growth assay and test of mitogenic activity of MST1/MSP

AlamarBlue was purchased from Life Technologies and used according to the manufacturer’s instructions. Cell cultures were incubated with alamarBlue for four hours at 37°C, after which the red fluorescence of the cellularly reduced resorufin was measured in the media with a plate reader at 555/570 nm. To determine the correlation between alamarBlue readings and cell number, passage one (P1) human tracheal basal cells were seeded in duplicate in 12-well dishes at 2500 cells/cm^2^. At days 1, 3, 5, and 7, alamarBlue readings were taken, after which the cell number was determined by staining cells with SYTO 60. Multiwell dishes were photographed on an inverted Olympus IX81 microscope with a 10× objective lens using a Rolera MGi Plus camera (QImaging) and MetaMorph software (Molecular Devices). The average number of cells in the field was determined from photographs of duplicate wells and was used as an approximation of cell growth. MST1/MSP was purchased from R&D Systems and was dissolved in PBS. For MST1/MSP growth assays, passage one (P1) human tracheal basal cells were seeded at 1000 cells/cm^2^ in triplicate in 6-well dishes. Positive control cultures were grown in LHC-9 medium and negative control cultures were grown in LHC-9 medium lacking 5 ng/ml EGF and 100 μg/ml BPE. Cells were fed with fresh medium and growth factor every other day beginning at day 1, at which point alamarBlue readings were also taken.

## Results

### Derivation of primary cultures of human tracheal basal cells

To examine expression of hundreds of cell surface receptors at a single cell level in human tracheobronchial basal cells, we chose a FACS-based approach using well characterized FACS antibodies, which would be more efficient than optimizing tissue staining for hundreds of antibodies. The basal cells were extracted from healthy adult human tracheal tissue (Figure 1A) using previously described methods [27,28,30,33]. Although our goal was to profile basal cell surface marker expression in as close to an *in vivo* setting as possible, we found that effective isolation of these cells from tissue required pronase digestion, which appeared to cleave some surface markers including the canonical basal cell marker CD44 [2,4]. Expression of CD44 and other surface markers by FACS was determined in individual live tracheal cells with the general gating strategy outlined in Additional file 3: Figure S1. Anti-CD44 FACS analysis of tracheal cell suspensions derived from fresh pronase-digested tissue identified fewer than the expected number of basal cells (predicted to be ~30% by previous studies [16,34] and empirically determined by us using anti-TP63 staining) (Additional file 7: Figure S2). Furthermore, the CD44-positive cells in these suspensions had a lower overall fluorescence intensity than control basal cells that had recovered *in vitro* and were removed with trypsinization (Additional file 7: Figure S2). To circumvent the pronase problem and increase the yield of cells for high-throughput surface marker profiling, we transiently expanded pronase-digested tracheal suspensions over 7–10 days without passaging. By potentially mimicking an injury state, this approach also had the advantage of possibly expanding subpopulations of basal cells that might be rare in a quiescent epithelium *in vivo*. During this time, tracheal cells were grown in a serum-free medium that is known to support tracheobronchial stem cell/progenitor activity [20,26]. After expansion, cells were removed with a mild trypsinization protocol, which we found cleaved few surface markers (see below and later).

**Figure 3:**
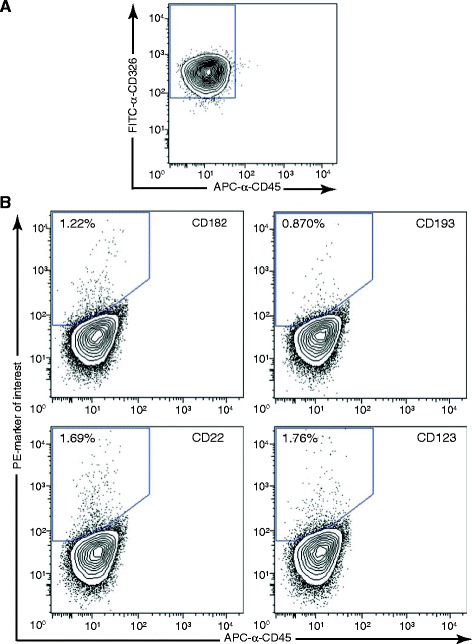
**Rare subpopulations of human tracheal cells are epithelial.** Primary human tracheal cells were triple stained for the epithelial-specific marker CD326/EpCAM (α-CD326-FITC), the hematopoietic-specific marker CD45 (α-CD45-APC), and the marker of interest (PE-conjugated). The epithelial cells in the triple-stained population were first identified by applying a CD326-positive/CD45-negative gate **(A)**. A PE-positive gate was created after compensation with the appropriate FMO controls to account for overlap between FITC and PE channels, and was applied to the CD326-positive/CD45-negative cells in the triple-stained populations shown in **(B)**. **(B)** Identification of PE-marker-positive cells in the CD326-positive/CD45-negative fraction of human tracheal cells.

The purity of the expanded primary tracheal cell cultures was first assessed by antibody staining for intracellular epitopes on canonical basal cell and differentiated columnar cell markers. 99% and 97% of the cells stained positive for the basal cell markers KRT5 and TP63, respectively (Figure 1B,C,K), while none of the cells stained positive for BTUB4, a ciliated cell marker [35], or MUC5AC and MUC16, tracheal mucinous cell markers [36,37] (Figure 1E-K). By FACS, 100% of the cells were positive for CD44 (Additional file 8: Figure S3A). These CD44-positive cells were not hematopoietic since none of the tracheal cells stained positive for the pan leukocyte CD45 marker [38] (Additional file 8: Figure S3B) using an anti-CD45 antibody clone that recognizes a trypsin-insensitive epitope [39]. In contrast, ~100% of cells from a control hematopoietic cell line were positively stained with the same anti-CD45 antibody (Additional file 8: Figure S3B). These data indicate that our primary tracheal cultures comprise only basal cells. KRT14, which is heterogeneously expressed in human and mouse large airway basal cells *in vivo* [11,13], was also expressed heterogeneously in the *ex vivo* cultures (Figure 1D,K) as reported in other *ex vivo* work [13], providing evidence for retention of some *in vivo* heterogeneity *ex vivo*.

### Verification of functional stem cell/progenitor activity in primary tracheal basal cell cultures

We next verified that our primary human tracheal basal cell cultures maintained functional stem cell/progenitor activity. It has been reported that with the isolation and expansion conditions we used, human tracheal basal cells retain multipotentiality, as revealed in rat tracheal xenograft assays [8]. In this assay, human tracheal basal cells are seeded into denuded rat tracheas and implanted as xenografts onto the backs of immunocompromised mice [8,30]. This assay is one of the most stringent assays for human tracheal stem cell activity. It allows for the generation of the most *in viv*o-like pseudostratified mucociliary epithelium, and it uniquely allows assessment of submucosal gland lineage potential [8]. To verify that our primary tracheal basal cell culture conditions maintain stem cell activity, we assessed the ability of basal cells derived from two different donor tracheas to repopulate denuded rat tracheas. After five weeks, engrafted primary tracheal basal cells gave rise to morphologically well-differentiated mucociliary epithelia and occasional submucosal glands (Figure 2). These epithelia and glands did not form in the absence of transplanted cells (Figure 2A), closely resembled their counterparts in normal human tracheal tissue (Figures 1A and 2B, C-E), and were of human origin, as assessed by FISH for a human-specific centromeric probe (Figure 2F). Thus, our isolation and culture conditions generally support the *ex vivo* expansion of tracheal basal cells capable of giving rise to multiple differentiated lineages.

**Figure 4:**
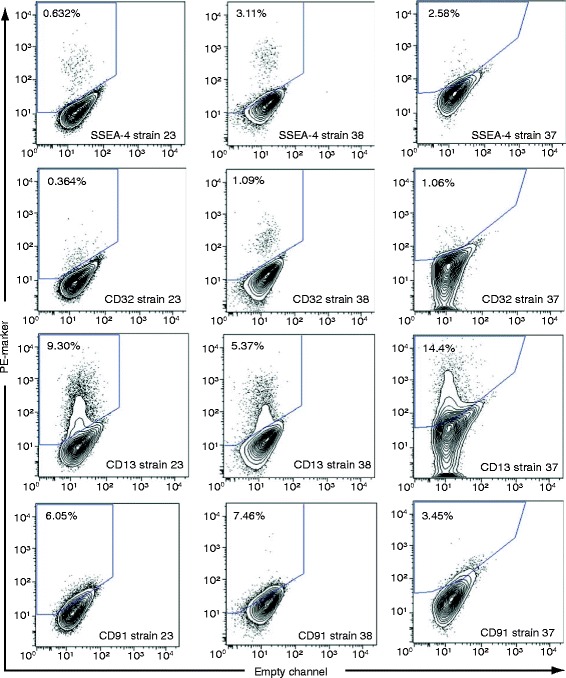
**Examples of FACS plots for cell surface markers that are heterogeneously expressed on tracheal basal cells.** Primary human tracheal basal cells were stained with PE-conjugated antibodies for the indicated markers. FACS staining for strains 23 and 38 were done at the same time using the same control unstained cells and therefore, have the same gates. FACS staining for strain 37 was done at a different time and the gates for this strain are drawn relative to the unstained controls used at that time.

### Comprehensive cell surface marker profiling identifies molecularly distinct subpopulations of human tracheal basal cells

To investigate expression of cell surface receptors at a single cell level and to identify new signaling pathways that potentially modulate tracheobronchial basal cell behavior, we used flow cytometry to profile expression of 332 surface markers in primary, non-passaged human tracheal basal cell cultures. Human tracheal basal cells derived from two different donor tracheas were stained and analyzed by FACS in a high-throughput manner. 314 out of the 332 tested antibodies yielded good quality FACS data in both basal cell strains (Additional file 2: Table S1). 108 of these antibodies stained less than 0.1% of the total population in both strains of basal cells, which we set as a cut-off for detectable expression (Additional file 2: Table S1). They provided a large number of isotype controls that indicated basal cells are not generally “sticky” to all antibodies. Some of these antibodies included anti-CD31, which is commonly used to detect endothelial cells [40], as well as three different anti-CD45 antibodies (Additional file 2: Table S1), and support our earlier findings that our tracheal cell cultures are not contaminated with non-basal cell types. 157 markers were detected in both basal cell strains and were expressed on varying numbers of cells (Table 2). These shared markers represented 80% and 95% of all expressed markers in each strain, indicating an overall strong similarity in the immuno-surface phenotype between the strains (Table 2). To further confirm the epithelial origin of rarer subpopulations of tracheal cells, we co-stained primary tracheal cell cultures with antibodies to a few of these markers, as well as CD45 and CD326/EpCAM. CD326 is only expressed in epithelial cells, including bronchial epithelial cells [41,42]. By gating on cells that were CD326-positive and CD45-negative, we confirmed that these rare subpopulations are indeed, epithelial (Figure 3).

**Table 1:** **Signaling receptors responding to secreted or cleaved factors that are expressed on human tracheal basal cells**

**Marker**	**Receptor**	**Ligand**	**Mean frequency of positive cells**
CD136	MST1R/RON	MSP	99.8
CD95	FAS	TNFSF6/FASLG	99.5
CD222	IGF2R	IGF2	99.0
CD221	IGF1R	IGF1	98.2
CD340	ERBB2	EGF	97.7
CD119	IFNGR1	IFNg	97.1
EGFR	EGFR	EGF	95.6
CD261	TNFRSF10A	TNFSF10/TRAIL	87.1
CD262	TNFRSF10B	TNFSF10/TRAIL	86.2
CD264	TNFRSF10D	TNF	66.4
CD271	NGFR	NGF, BDNF, NT-3, NT-4	53.9
CD130	IL6ST (coreceptor for IL6R, LIFR)	IL6, LIF	43.6
CDW218a	IL18R1	IL18	22.5
CD181	CXCR1	IL8	19.2
CD132	IL2RG (coreceptor for IL7RA, IL9R)	IL17A, IL9	18.7
CD295	LEPR	Leptin	10.0
CD117	CKIT	SCF	3.79
CDW198	CCR8	TARC, MIPb	2.23
CD193	CCR3	CCL11, CCL26, CCL7, CCL13, CCL5	1.84
CD126	IL6R	IL6	1.49
CD266	TNFRSF12A	TNFSF12	1.08
CD304	NRP1	SEM3A, VEGF-165, PLGF-2	1.01
CD195	CCR5	MCP2, MIP-1a, MIP-1b, CCL5	0.656
CD182	CXCR2	IL8	0.643
CD218b	IL18RAP	IL18	0.557
CD120b	TNFRSF1B	TNFSF2/TNFa, TNFSF1/LTA	0.537
PAC1	ADCYAP1R1	PACAP-27, PACAP-38	0.517
CD129	IL9R	IL9	0.459
CD184	CXCR4	CXCL12	0.447
CD120a	TNFRSF1A	TNFSF2/TNFa, TNFSF1/LTA	0.318
CD217	IL17RA	IL17A	0.232
CD309	KDR/VEGFR2	VEGF	0.196
CD118	LIFR	LIF	0.169

Potential ligands for the receptors were assigned using information at www.genecards.org. Mean frequencies of positive cells are from Additional file 2: Table S1.

**Figure 5:**
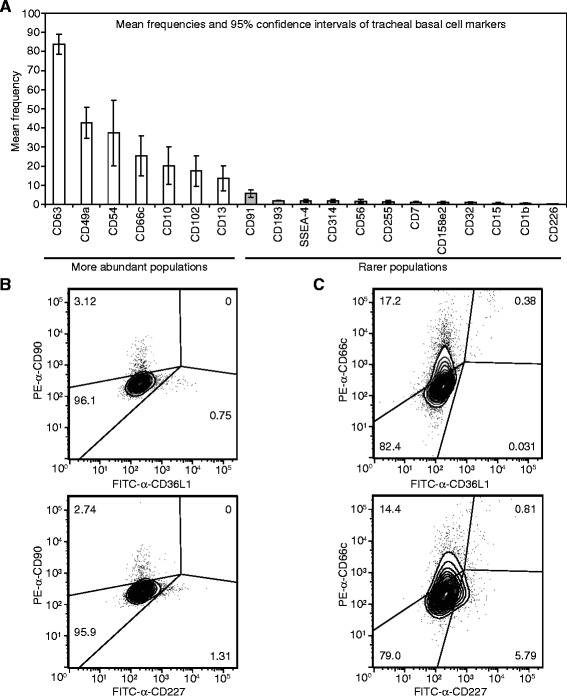
**Subpopulations of tracheal basal cells vary in their abundance. (A)** Histogram showing subpopulations with overlapping and non-overlapping 95% confidence intervals of mean frequency in the total population. Data are from Additional file 6: Table S2 and represent a subset of markers. **(B, C)** Analysis of potential dual expression of a subset of heterogeneously expressed markers in tracheal basal cells. Primary human tracheal basal cells were stained with FITC and PE-conjugated antibodies for the indicated markers. Gates were created after compensation with the appropriate FMO controls to account for overlap between FITC and PE channels.

We then chose to validate 110 of the shared expressed markers in one or two additional basal cell strains derived from different donor tracheas (Additional file 2: Table S1). These validation strains did not have detectable contamination with CD31 or CD45-positive cells (Additional file 2: Table S1). 105 of the 110 basal cell markers were detected in these additional strains, identifying a large set of markers expressed in three to four independently isolated basal cell strains. The high degree of validation (95%) suggests that most of the markers identified as being shared between the initial screening strains can be extended to all isolates of human tracheal basal cells. Within the group of 105 markers, 74 were expressed with a mean frequency of less than 90% (Additional file 6: Table S2), suggesting that they are heterogeneously expressed within the basal cell bulk population. Heterogeneous expression of these markers (especially low expression) was not due to our mild trypsinization protocol. Prior control studies indicated that the anti-CD31 and anti-CD45 antibodies, as well as most of the antibodies to the heterogeneously expressed markers recognize trypsin-insensitive epitopes [31] (summarized in Additional file 1: Table S3). In these control experiments, a cocktail of collagenase, hyaluronidase, dispase, and a two-fold higher concentration of trypsin only affected FACS detection of 17 of 68 tested markers of heterogeneity. Furthermore, of these 17 markers, detection of only three (CD62L, CD195, CD213a2, 4.4% of number assayed) was affected by trypsin in isolation. Since expression of these three markers was only affected two to three-fold while their mean frequencies in the bulk basal cell population ranged from 0.6-3.7%, even partial sensitivity to trypsin cannot account for their heterogeneous expression. For 47 markers, mean frequencies were measured between 0.1 to <90% of the total basal cell population with 95% confidence (Additional file 6: Table S2), identifying good candidates for heterogeneously expressed markers. Examples of representative FACS plots for these markers are shown in Figure 4. Some markers displayed a lot of variation in the number of positive cells in different basal cell isolates, while others, especially those expressed on fewer than 6% of all basal cells, were detected at consistently similar frequencies (Additional file 6: Table S2). By looking at marker frequencies with non-overlapping 95% confidence intervals, it appeared that using several different cut-offs, subpopulations of basal cells could be grouped into more (e.g. ~ > 13%) or less (e.g. ~ < 6%) abundant subpopulations (see Figure 5A for examples).

**Figure 6:**
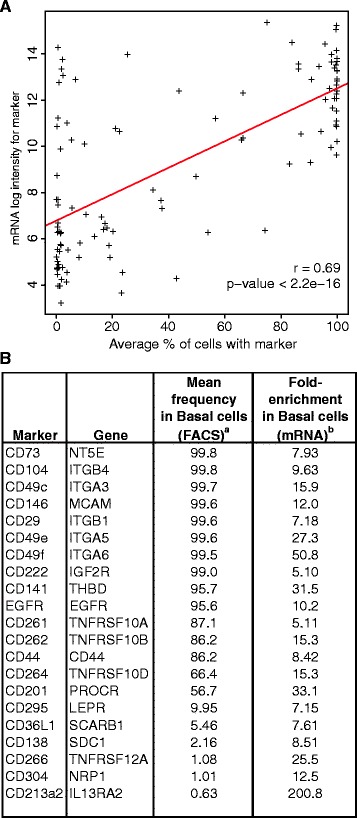
**Comparison of large airway basal cell marker expression by FACS and mRNA analysis. (A)** Scatter plot comparing the frequency of cell surface marker expression with corresponding marker mRNA expression in bulk population basal cells. The average percentage of marker expression was taken from Additional file 6: Table S2 and mRNA data were from Hackett NR et al., *PLoS One*, 2011. The mRNA expression data represent the average of five replicate microarray hybridizations, with the value for the most highly expressed probe for a given gene being plotted. The red line shows the linear relationship between the two properties (Pearson correlation r = 0.67; t = 7.85; p-value < 0.001). **(B)** Table of marker genes that were determined to be more than 5-fold enriched at the mRNA level in basal cells relative to other large airway epithelial cells. mRNA data are from Hackett NR et al., *PLoS One*, 2011, and are from the most highly expressed probe for the gene.

**Figure 7:**
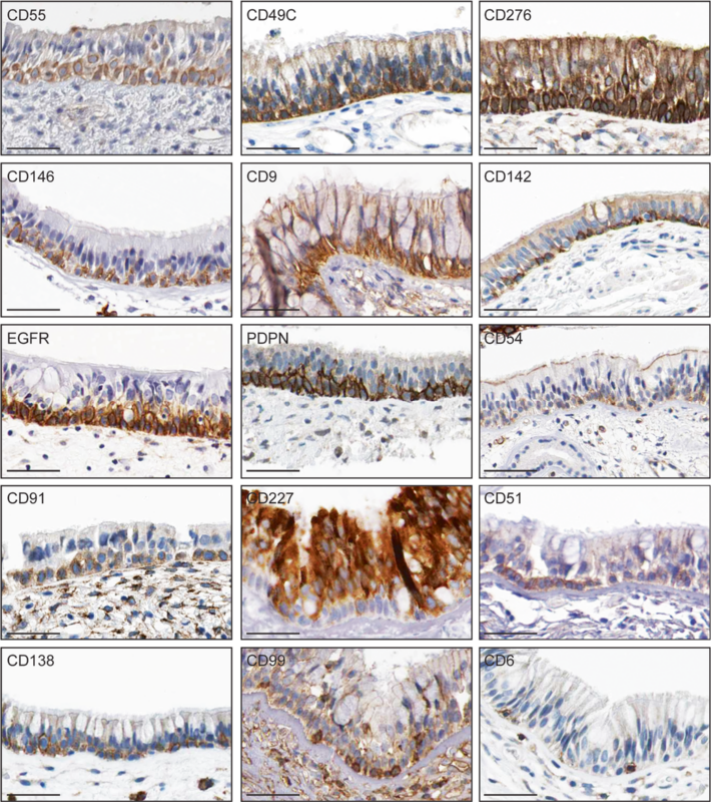
**Expression of a subset of FACS-identified cell surface markers** ***in vivo*** **.** Human nasopharyngeal (CD55, CD49C, CD276, CD146, EGFR, PDPN, CD91, CD138, CD54, CD227, CD99, CD6) or bronchial (CD9, CD142, CD51) tissue was stained with the indicated antibodies. Images are from The Human Protein Atlas online resource (www.proteinatlas.org). Scale bars are 50 μm.

While a complete analysis of the total number of subpopulations represented by marker heterogeneity was beyond the scope of this work, we, nevertheless, investigated potential overlap between some subpopulations. CD90-expressing cells (~3% of the total population) did not overlap with CD36L1 (0.75%) or CD227 (1.31%)-positive cells, indicating that CD90-positive cells are distinct from these other subpopulations (Figure 5B). Furthermore, CD36L1 and CD227 did not mark identical subpopulations since >90% of CD36L1, but only 12% of CD227-positive cells expressed CD66c (Figure 5C).

### Microarray corroboration of basal cell surface marker expression

To corroborate the surface marker heterogeneity observed by FACS by another method, we investigated if marker FACS data correlated with marker mRNA expression in the bulk population. We focused on the 105 surface markers that were validated in three or more basal cell isolates (Additional file 6: Table S2), as well as 14 additional signaling receptors that were not analyzed by FACS beyond the initial high-throughput screening (part of Table 1). mRNA levels were determined from five microarray datasets of gene expression in primary human bronchial basal cells that were grown under the same culture conditions used by us [18]. Probes for 111/119 markers were on the microarrays yielding 92% coverage. Overall, we found that the percentage of marker-positive cells detected by FACS was correlated with mRNA expression for that marker (Figure 6A). These data corroborate the accuracy of the antibodies in detecting their antigens, and support the FACS data indicating basal cells exist as distinct, molecularly identifiable subpopulations. We also found that in the microarray analysis [18], 21/111 of our markers were independently annotated as basal cell-enriched genes relative to the other large airway epithelial cells (Figure 6B). These data additionally suggest that a number of our markers are not only expressed on basal cells, but also define basal cell lineage-specific markers.

**Table 2:** **Overlap of marker expression in primary strains of human tracheal basal cells derived from different tracheas**

**Frequency of positive cells (%)**	**Total markers**			**Overlap of markers between strains**		
	**Strain 23**	**Strain 38**	**Validation strains** ^**a**^	**Strain 23** ^**b**^	**Strain 38** ^**c**^	**Validation strains** ^**a**^
<0.1	147	117		108 (73%)	108 (92%)	
0.10-0.99	62	87		40 (65%)	40 (46%)	
1.0-9.9	34	41		23 (68%)	23 (53%)	
10.0-100	70	68		66 (94%)	66 (97%)	
0.1-10.0	96	128		85 (89%)	85 (66%)	
0.1-100	166	196	111	157 (95%)	157 (80%)	105 (95%)

^a^Validation data were obtained using multiple strains of primary human tracheal basal cells that were not used in the initial high-throughput screens. ^b^Number and (percentage) of markers from strain 38 that are expressed (or not expressed, <0.1%) in the same indicated category of strain 23. ^c^Number and (percentage) of markers from strain 23 that are expressed (or not expressed, <0.1%) in the same indicated category of strain 38.

**Figure 8:**
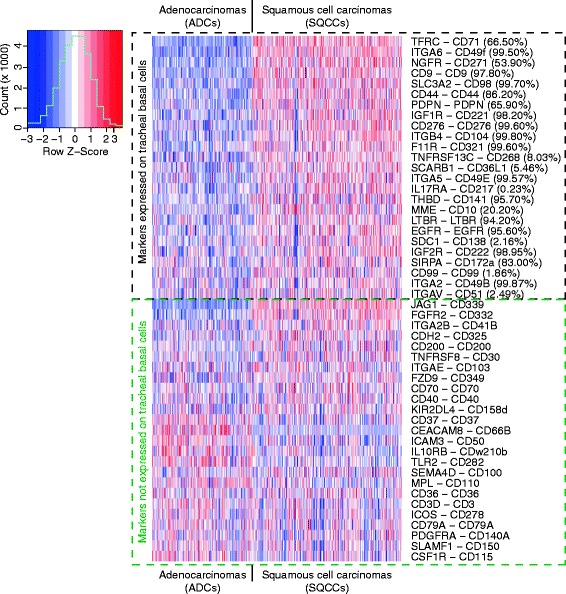
**Enrichment of basal cell surface markers in lung squamous cell carcinomas.** Heat map of mRNA expression in lung adenocarcinomas (ADCs) and squamous cell carcinomas (SQCCs) for the top 25 SQCC-enriched markers from those detected (Additional file 4: Table S4) or not detected (Additional file 5: Table S5) on tracheal basal cells by FACS. For each marker, the normalized log RSEM value for mRNA expression in each sample was scaled according to the distribution across samples. The percentage shown beside each marker name indicates the mean frequency of marker detection in bulk population tracheal basal cells (from Additional file 2: Table S1).

### *In vivo* expression of basal cell surface markers in normal airway tissue

**Figure 9:**
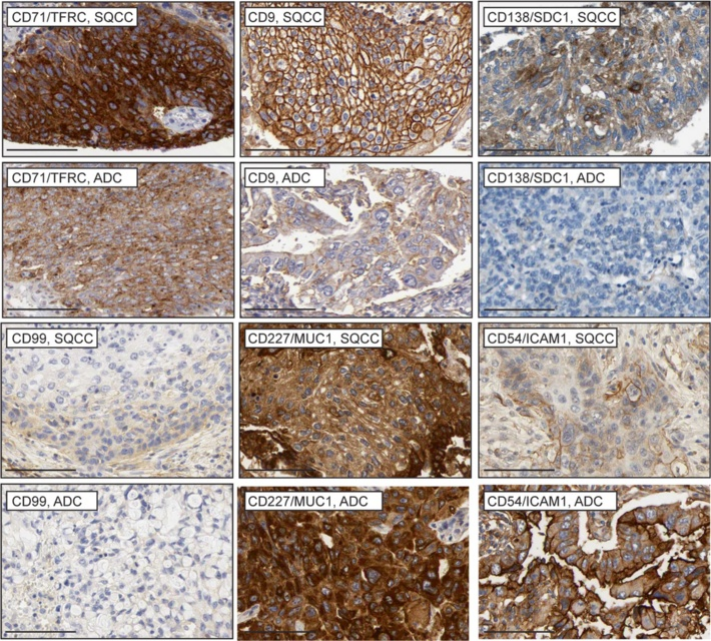
**Protein expression of basal cell surface markers in lung cancer.** Images are from the Human Protein Atlas online resource (www.proteinatlas.org). Quantification of staining is shown in Additional file 9: Table S6. Scale bars are 100 μm.

To determine if some of the basal cell markers we identified *in vitro* might mark basal cells in normal airway tissue *in vivo*, we used the Human Protein Atlas (www.proteinatlas.org), which is an expansive online resource for normal and cancer tissue immunostaining for the human proteome [43-47]. Normal tissues included the nasopharynx and bronchus, which share the same type of epithelium as the trachea. Good tissue sections were available for 83 of the 105 markers we identified in three or more basal cell isolates. 63 of these markers showed some expression in airway epithelia. Expression ranged from all epithelial cells to exclusively all basal cells, to only subsets of basolaterally located cells (but not necessarily, basal epithelial cells). By using the traditional morphological criteria that were used to establish basal cell-specific expression of the canonical basal cell markers (cuboidal shape and occupying exclusively and uniformly basolateral positions) KRT5, KRT14, CD44, and GSA-I-B4 reactivity [3,4,48-50], it appeared that CD55, CD49C, CD276, CD146, CD9, CD142, and the EGFR, which were detected on most basal cells *in vitro*, were also expressed on most basal cells *in vivo* (Figure 7). While PDPN, CD91, CD138, and possibly CD51, also appeared to be enriched on most basal cells *in vivo* (Figure 9), *in vitro*, these markers were expressed only on subsets of basal cells. Collectively, these eleven markers identify pan-basal cell lineage markers. CD54, CD227, CD99, and possibly CD6, were expressed heterogeneously *in vitro* and at basolateral positions *in vivo* (Figure 7). However, in these cases, it is not clear if they mark subsets of basal cells or rare, infiltrating other cell types.

### *In vivo* expression of basal cell surface markers in lung squamous cell carcinomas

To further support the *in vivo* relevance of the new basal cell markers and to begin to explore their potential relevance to cancer biology, we also examined marker expression in squamous cell lung cancers (SQCCs), which likely arise from basal cells. SQCCs develop in the large airways and >90% of SQCCs express pan-basal cell lineage markers such as KRT5 and TP63 [51,52]. To strengthen the interpretation that some SQCC marker expression reflects the basal cell origin of SQCCs, we also examined marker expression in control lung adenocarcinomas (ADCs), most of which are not likely to arise from basal cells. ADCs tend to develop distally, with KRT5 and TP63 expression being infrequent [51,52]. For this analysis, we used the same panel of 119 markers that was employed in the microarray analysis. 110 of these markers could be mapped to genes in The Cancer Genome Atlas lung cancer RNAseq datasets [32]. 22% (24/110) of these markers had significantly higher expression (p-value < 0.05) in SQCCs compared to ADCs (Additional file 4: Table S4, Figure 8). This degree of enrichment is much larger than expected by chance, as using the same statistical analysis and threshold for significance (p-value < 0.05), only 1.4% (290/20,725) of total genes was estimated to be significantly upregulated in SQCCs relative to ADCs. The degree of basal cell marker enrichment in SQCCs was also greater than observed for 101 control markers not expressed on basal cells (expressed on <0.1% of all basal cells) (7%, Additional file 5: Table S5, Figure 1). In this latter case, the 7% enrichment is likely an overestimate of true enrichment due to the misclassification of some markers as being not expressed on basal cells. Some FACS antibodies may have yielded false negative results due to their inability to recognize all protein isoforms and their modified variants for a given gene. Notably, among the control markers, the two most SQCC-enriched proteins, JAG1 and FGFR2, were not detected on basal cells by FACS, but by mRNA and other protein analyses, are known to be expressed on human airway basal cells growing *in vitro* and *in vivo* [18,53-55].

While the fold-differences in marker mRNA expression between the SQCCs and ADCs were not dramatically different, many of these differences are likely to be meaningful at the protein level. Protein expression of 14/24 of the SQCC-enriched markers has been detected in SQCCs [56-67], and at least in seven cases (CD98/SLC3A2, CD44, CD221/IGF1R, CD276, CD141/THBD, EGFR, PDPN), marker protein expression was reported to be greater or more common in SQCCs as compared to ADCs [58,62-66,68-70]. Furthermore, expression of three markers has been reported to be prognostic with regards to overall survival in SQCCs (CD98/SLC3A2, CD44, PDPN) [60,63,71-73]. To obtain protein data to corroborate the mRNA data for the remaining SQCC-enriched markers not previously interrogated in SQCCs, we examined marker protein expression in lung cancer samples through the Human Protein Atlas (www.proteinatlas.org). While the small number of cancer samples did not allow assessment of statistical significance, several of the new markers identified through mRNA analysis also showed trends of increased protein expression in SQCCs relative to ADCs (CD71/TFRC, CD9, PDPN, CD138/SDC1, CD99) (Figure 9, Additional file 9: Table S6). Using the Human Protein Atlas, we also found that some of the heterogeneously expressed basal cell markers that were not identified as SQCC-enriched markers, were, nevertheless, still expressed in SQCCs (Figure 9, Additional file 9: Table S6, CD227/MUC1, CD54/ICAM1).

**Figure 10:**
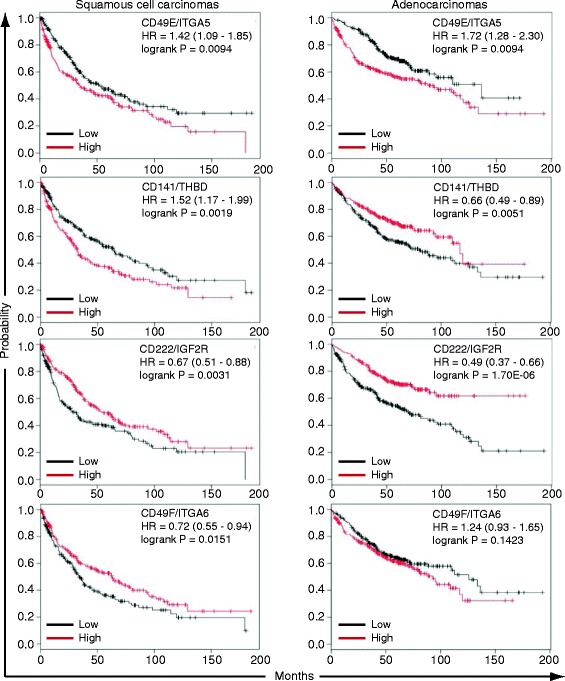
**Prognostic significance of basal cell markers enriched in lung squamous cell carcinomas (SQCCs) and adenocarcinomas (ADCs).** Univariate Kaplan-Meier survival analysis was performed using the entire lung SQCC (n = 421) and ADC (n = 487) datasets compiled at KM-plotter (http://kmplot.com/analysis/) [74]. HR = Hazard ratio (95% confidence interval). HR > 1.00 = poor overall survival. HR < 1.00 = better overall survival. Affymetrix probes were 201389_at (CD49E/ITGA5), 203887_s_at (CD141/THBD), 201393_s_at (CD222/IGF2R), and 215177_s_at (CD49F/ITGA6).

Since some of the SQCC-enriched markers have already been shown to have prognostic significance to SQCCs [60,63,71-73], we examined the remainder of these markers for the relationship of their expression to overall survival by Kaplan Meier survival analysis. We used KM-plotter (http://kmplot.com/analysis/) [74], an online resource that compiles gene expression and overall survival data from the Gene Expression Omnibus (http://www.ncbi.nlm.nih.gov/geo/) and The Cancer Genome Atlas (http://cancergenome.nih.gov/). Using the entire SQCC and ADC datasets, we found that expression of four markers had significant associations with overall SQCC patient survival (Figure 10). Two markers were associated with worse overall survival (CD49E/ITGA5, CD141/THBD) and two markers were associated with better overall survival (CD49F/ITGA6, CD222/IGF2R). For CD141/THBD and CD49F/ITGA6, the direction of the association with overall survival was unique to SQCCs. This finding could relate to these markers having different functions in SQCCs and ADCs and their distinct cells of origin. CD49E/ITGA5 and CD222/IGF2R were prognostic in the same direction in both SQCCs and ADCs, suggesting that despite elevated expression in SQCCs, these proteins may perform similar functions in the two tumor types and their cells of origin. Collectively, these normal and tumor tissue data support the relevance of our newly identified markers to *in vivo* populations of normal and transformed basal cells.

### MST1/MSP stimulates proliferation of tracheal basal cells

Among the surface markers detected on human tracheal basal cells were 33 receptors that are involved in signaling by cleaved or secreted factors (Table 1). Receptors for EGF, IGF, insulin, NGF, MST1/MSP, IFNγ, FASL, and some TNF family growth factors were expressed on most basal cells while receptors for leptin, LIF, VEGF, SCF, chemokines, interleukins, and other TNF family members were expressed on fewer cells. These observations suggest that some factors may act on all basal cells while others may regulate distinct subpopulations. Some of these receptors are known to be functional as EGF and insulin are essential components of the serum-free medium used to culture human tracheal basal cells [19,20,22] and NGF promotes survival of basal cells after RSV infection [75].

**Figure 11:**
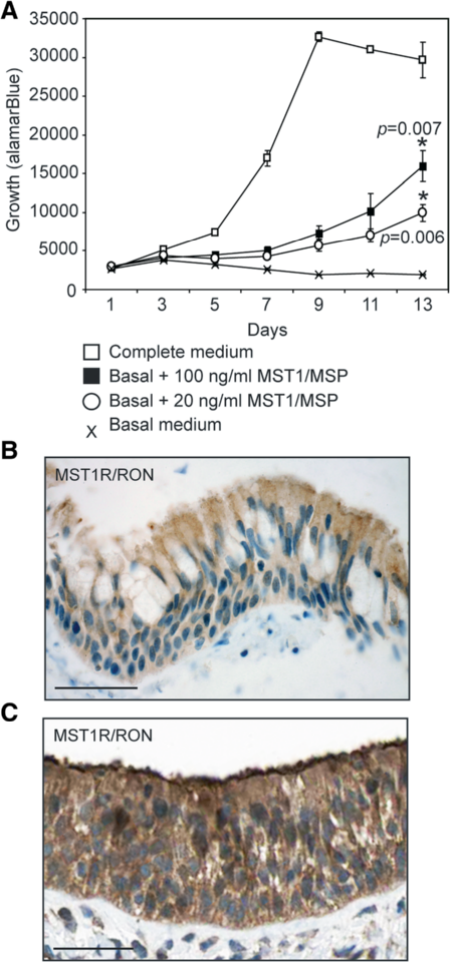
**MST1/MSP is a mitogen for tracheal basal cells and MST1R/RON is expressed in all large airway basal cells** ***in vivo*** **. (A)** The MST1R/RON ligand MST1/MSP promotes growth of human tracheal basal cells. Growth was measured by the alamarBlue assay. Each data point represents the mean of triplicate cultures with the standard deviation shown. *p* values were calculated for the day 13 data point relative to cells growing in control basal medium without EGF and BPE, using a two-tailed student’s *t* test. **(B)** Human tracheal tissue stained with anti-MST1R/RON (Santa Cruz). **(C)** Human nasopharyngeal tissue stained with anti-MST1R/RON. Image is from The Human Protein Atlas online resource (www.proteinatlas.org). Scale bars are 50 μm.

Since the biological function of most of these receptors is unknown in large airway basal cells, we chose to investigate one receptor in more detail. CD136/MST1R/RON is the receptor for MST1/MSP and by FACS, was expressed on all basal cells (Table 1, Additional file 2: Table S1). Although a biological function for MST1/MSP has not been reported in basal cells, it has previously been shown to directly bind cultured human tracheal basal cells and to induce phosphorylation of MST1R/RON [76]. Furthermore, MST1R/RON missense mutations and expression of an alternatively spliced oncogenic MST1R isoform have been reported in lung SQCCs [32,77], suggesting this receptor may promote growth of basal cells, as it does in TP63-positive keratinocytes [78]. To address this possibility, we cultured tracheal basal cells in the absence of EGF and bovine pituitary extract, which allowed the cells to remain viable, but reduced cell growth. We then added two different doses of MST1/MSP and measured cell growth by alamarBlue, which we found is directly proportional to cell number (Additional file 10: Figure S4). Both doses of MST1/MSP promoted growth, with the higher dose yielding half the maximal cell number obtained with optimized amounts of EGF and bovine pituitary extract (Figure 11A). We also verified that MST1R/RON was expressed in tracheal basal cells *in vivo* by immunostaining human tracheal tissue with anti-MST1R antibodies. MST1R/RON expression was detected in all cells of the tracheal epithelium, including basal cells (Figure 11B). This expression pattern was corroborated by the Human Protein Atlas with a different anti-MST1R/RON antibody in the nasopharyngeal epithelium (Figure 11C), where MST1/MSP has been reported to increase the beat frequency of ciliated cells [76]. Thus, MST1/MSP has distinct effects on basal and non-basal cell types in the large airways.

## Discussion

A number of studies have provided evidence for basal cell heterogeneity in the large airways [3,6,8-12,79], but there has been little phenotypic description of this heterogeneity at a molecular level. The goal of this work was to specifically investigate heterogeneity among basal cells using cell surface markers that can help identify/purify subpopulations and reveal signaling pathways that regulate their behavior. To this end, we identified 105 cell surface markers that were expressed on varying percentages of basal cells isolated from three or more healthy tracheas. Furthermore, the 95% validation rate of markers detected on the initial two screening strains suggests that we may have identified as many as 157 surface markers for large airway basal cells. With 95% confidence, 47/105 validated markers were expressed on between 0.1 to <90% of all basal cells, identifying good candidates for heterogeneously expressed markers. This interpretation of the FACS data was independently supported by microarray gene expression profiling of large airway basal cells. Here, we found that mRNA expression for most markers was proportional to our measurements of the abundance of marker-positive cells in the bulk basal cell population. However, the total number of distinct subpopulations is not clear. At a minimum, subsets of cells can generally be grouped into more (e.g. >13%) or less (e.g. <6%) abundant subpopulations. Our analysis of a small set of markers indicate some subpopulations are distinct (e.g. CD90 from CD36L1 and CD227), while some overlap (e.g. CD66c and CD36L1). Ultimately, extensive multi-parametric flow cytometry will be necessary to refine the subclassification of basal cell subpopulations. The recently developed mass spectrometry-coupled single cell flow cytometer, in which up to 30 parameters can be simultaneously visualized [80], could rapidly deconvolute the complex heterogeneity observed in our experiments.

The origin of the observed surface marker heterogeneity is not clear. Some markers showed substantial variations in expression between different basal cell isolates, which could simply reflect highly variable stochastic responses to *ex vivo* culturing. Alternatively, they could reflect pre-existing differences in the numbers of these subpopulations between different donor tracheas, which might be influenced by age (range was 38–59) or other factors (although all donors were non-smokers). However, many markers, especially those expressed on <6% of all cells, were consistently detected at similar frequencies in all tested basal cell isolates, suggesting that some subpopulations are under tight control. This might reflect the partitioning of some basal cells into more stem/progenitor-like cells as has been previously suggested [8,10,13]. Indeed, in co-culture assays, human colony forming progenitors have been estimated between 0.15-0.6% of all basal cells, while in semi-solid media, the frequencies have been measured between 0.4-12% [5,9,10]. Consistent with this possibility, CD338/ABCG2 was found on 0.12-0.15% of tracheal basal cells (Additional file 2: Table S1). This marker is the major transporter that is responsible for the Hoechst 33342 efflux properties that define “side population” (SP) progenitor cells [81]. Similar to our findings, previous work reported that 0.12% of human tracheal epithelial cells are ABCG2/TP63-double positive side population cells, but additionally found that these cells might have enhanced progenitor activity [82]. Another one of our markers, CD117/KIT, was reported to mark embryonic-like multipotential human lung stem cells and has been reported to be co-expressed with TP63 in rare bronchiolar cells *in vivo* [83].

Most of the heterogeneously expressed markers have no prior association with normal epithelial stem cells. However, CD66c/CEACAM6 was reported to be a marker of colorectal cancer stem cells [84] and was also shown to be heterogeneously expressed in cultured human large airway basal cells by confocal microscopy [85]. Many other markers (CD54, CD182/CXCR2, CD193/CCR3, CD90, CD13) have been reported to be expressed on mesenchymal stem cells (MSCs) and are often co-expressed with markers such as CD166, CD29, CD44, CD73, and CD49B, which were detected on most tracheal basal cells [86-94]. Given the greater than 99% KRT5 positivity of our cultured tracheal cells, our cultures are not likely contaminated with typical MSCs. Instead, we believe that some surface markers are shared between certain mesenchymal and tracheal epithelial stem/progenitor cell populations. Indeed, some liver progenitors are marked with both MSC and epithelial markers [88,89].

*In vivo* validation of all of the markers will be challenging. In general, antibody staining of tissue is more problematic than FACS staining, and many markers were detected on rare cells, which can be difficult to distinguish from hematopoietic cells in tissue, but could be excluded in our *in vitro* studies. Furthermore, the dramatic differences between the *in vivo* and *in vitro* environments may affect the steady state levels of certain subpopulations and expression of some markers. In the normal *in vivo* airway epithelium, basal cells are mostly quiescent and form many adhesive contacts with each other and a complex extracellular matrix. *In vitro*, our basal cells were characterized while proliferating subconfluently on a two-dimensional extracellular matrix. Nevertheless, we confirmed that eleven markers identified *in vitro* were strongly enriched in the entire large airway basal cell compartment *in vivo*, revealing pan-basal cell lineage markers. Seven of these eleven markers were expressed on most basal cells *in vitro* (CD55, CD49C, CD276, CD146, CD9, CD142, EGFR), suggesting that their expression is not greatly influenced by the environment or the proliferative state. Four of these markers (PDPN, CD91, CD138, CD51) were much more heterogeneously expressed *in vitro* than *in vivo*. PDPN, CD138/SDC1, and CD51/ITGAV are adhesion molecules and might be downregulated *in vitro* because of the 2D growth conditions, which lack a 3D matrix. Interestingly, PDPN has been reported to be heterogeneously expressed among TP63 and CD44-positive cells in lung SQCCs [63]. Thus, absence of PDPN expression is more commonly observed under conditions of growth (normal basal cells *in vitro* and transformed basal cells in lung SQCCs) rather than quiescence (basal cells in healthy large airways). It is, therefore, possible that PDPN expression may mark a more quiescent basal cell state, which could explain its correlation with better prognosis in lung SQCCs [63,71,73]. However, the function or regulation of PDPN may differ between distinct types of basal cells since in SQCCs derived from other tissues, PDPN expression is correlated with poor prognosis [95,96]. Although some of the markers that were heterogeneously expressed *in vitro* were also heterogeneously expressed *in vivo* (e.g. CD54, CD227, CD99, CD6), it is unclear if these *in vivo* populations are basal epithelial cells or hematopoietic cells.

By comparing marker expression in lung squamous cell carcinomas (SQCCs) and adenocarcinomas (ADCs), we obtained further evidence for the *in vivo* relevance of some of the markers, as well as the basal cell origin of SQCCs. We identified 24 basal cell markers that were significantly enriched in SQCCs relative to ADCs. Many of these markers additionally represent new and potentially useful biomarkers for SQCCs. Based on our *in vitro* studies, some of these markers appear to be pan-basal cell lineage markers or are at least associated with a proliferative basal cell state. Accordingly, some of the SQCC-enriched basal cell markers might read-out or contribute to basal cell functions that promote malignancy (e.g. proliferation). Alternatively, some of the markers might be remnants of the normal basal properties that oppose a cancerous state (e.g. adhesion). Consistent with both possibilities, CD98/SLC3A2 expression was previously found to be associated with shorter overall survival, while CD44 and PDPN expression was correlated with longer overall survival [60,63,71-73]. In addition to these markers, we found that expression of CD49E/ITGA5 and CD141/THBD was also associated with shorter overall survival, while expression of CD222/IGF2R and CD49F/ITGA6 was associated with longer overall survival. Interestingly, in ADCs, CD141/THBD expression was actually associated with longer overall survival, while CD49F/ITGA6 was not prognostic. These data suggest that CD141 and CD49F may have distinct cell of origin and disease-specific functions.

Some of the SQCC-enriched markers were only detected on subsets of normal basal cells cultured *in vitro* (CD268/TNFRSF13C, CD36L1/SCARB1, CD217/IL17RA, CD10/MME, CD138/SDC1, CD99, CD51/ITGAV) and possibly found *in vivo* (CD51, CD99). These data suggest that the normal subpopulations defined by these markers might specifically be involved in SQCC pathogenesis. Two markers of normal heterogeneity (CD54 and CD99) also appeared to be heterogeneously expressed in SQCCs. In SQCCs, CD99 expression seemed restricted to less well-differentiated, basal-like cells. The intratumoral heterogeneity of expression of these markers could be relevant to the cellular hierarchical organization that has been described in SQCCs with regards to tumor initiating activity [97,98].

Finally, besides being molecular markers of phenotypic diversity, many of the surface antigens we detected provide new insight into signaling pathways that may regulate basal cell behavior. This information will be important to understand how large airway progenitor self-renewal and differentiation are regulated, how basal cell homeostasis may be deregulated during disease pathogenesis, and to ensure subpopulations with distinct growth factor requirements can be optimally cultured *ex vivo*. As a testament to the potential importance of these signaling pathways, we found that the MST1R/RON receptor tyrosine kinase was expressed on all tracheal basal cells and that its ligand, MST1/MSP promoted basal cell growth. This function of MST1/MSP contrasts with its non-proliferative ability to enhance beat frequency of ciliated cells [76], but is in agreement with its growth stimulatory role in keratinocytes [78], suggesting a potentially conserved mitogenic role in TP63-positive basal cells. This mitogenic function of MST1/MSP could also explain the expression of oncogenic splice variants of MST1R/RON in SQCCs and the occurrence of rare MST1R missense mutations in these cancers [32,77].

## Conclusions

We have identified 105 cell surface markers that are expressed on cultured primary human tracheal basal cells, and we provide evidence for 47 of these markers being heterogeneously expressed in the bulk population. Eleven markers appear to be pan-basal cell lineage markers *in vivo* and 24 markers are enriched in lung SQCCs relative to ADCs, with four of these markers having prognostic relevance to lung cancer. We also show that some of these newly identified markers are functional in basal cells, as MST1/MSP, the ligand for CD136/MST1R/RON, is a mitogen for basal cells. Overall, this study provides evidence for molecularly distinct heterogeneity within the human tracheal basal cell population, and it identifies many new opportunities for the directed investigation of stem cells/progenitors in the normal tracheobronchial epithelium and in SQCCs.

## Additional files

Additional file 1: Table S3. Lack of trypsin sensitivity of most markers that are heterogeneously expressed on human tracheal basal cells. This table summarizes the sensitivities (or lack thereof) of various cell surface markers to trypsin digestion. Additional file 2: Table S1. Cell surface marker expression in multiple independently-derived strains of primary human tracheal basal cells. This table summarizes all of the FACS data for the manuscript. Additional file 3: Figure S1. General gating strategy for all flow cytometric analyses (FACS) of human tracheal basal cells. This figure presents the general gating strategy used to identify marker expression by FACS in individual, viable human tracheal basal cells. Additional file 4: Table S4. Analysis of lung squamous cell carcinoma enrichment of mRNA expression of surface markers detected on tracheal basal cells. This table summarizes the degree of enrichment of basal cell marker expression in patient lung SQCCs relative to ADCs using TCGA RNA-seq data. Additional file 5: Table S5. Analysis of lung squamous cell carcinoma enrichment of mRNA expression of surface markers not detected on tracheal basal cells. This table summarizes the degree of enrichment of expression of markers not detected on tracheal basal cells by FACS, in patient lung SQCCs relative to ADCs using TCGA RNA-seq data. Additional file 6: Table S2. Mean frequencies of potential human tracheal basal cell subpopulations and their variations between different primary basal cell strains. This table summarizes the most reproducibly detected cell surface markers and statistical analysis. Additional file 7: Figure S2. Pronase digestion reduces CD44 expression in tracheal basal cells. This figure presents FACS and immunostaining data that support pronase digestion cleaving epitopes on CD44. Additional file 8: Figure S3. Primary human tracheal epithelial cell cultures consist entirely of CD44+ CD45- cells. This figure presents FACS data that indicate primary tracheal cell cultures are entirely comprised of CD44-positive and CD45-negative cells. Additional file 9: Table S6. Summary of IHC staining of lung cancer tissues from the Human Protein Atlas (www.proteinatlas.org). This table presents the number of samples examined and the quantitative assessment of marker expression. Additional file 10: Figure S4. AlamarBlue readings are directly proportional to tracheal basal cell number. This figure presents a standard curve that demonstrates a linear relationship between alamarBlue readings and tracheal basal cell number.

## Abbreviations

FACS: Fluorescence activated cell sorting
SQCC: Squamous cell carcinoma
ADC: Adenocarcinomas
TCGA: The Cancer Genome Atlas
