# Supplementary material for: Cell surface marker profiling of human tracheal basal cells reveals distinct subpopulations, identifies MST1/MSP as a mitogenic signal, and identifies new biomarkers for lung squamous cell carcinomas
Source: Respir Res. 2014 Dec 31;15(1):160. doi: 10.1186/s12931-014-0160-8 (PMC4343068; doi:10.1186/s12931-014-0160-8)
Supplement: Additional file 3: Figure S1. — General gating strategy for all flow cytometric analyses (FACS) of human tracheal basal cells. This figure presents the general gating strategy used to identify marker expression by FACS in individual, viable human tracheal basal cells. [file 12931_2014_160_MOESM3_ESM.pdf]

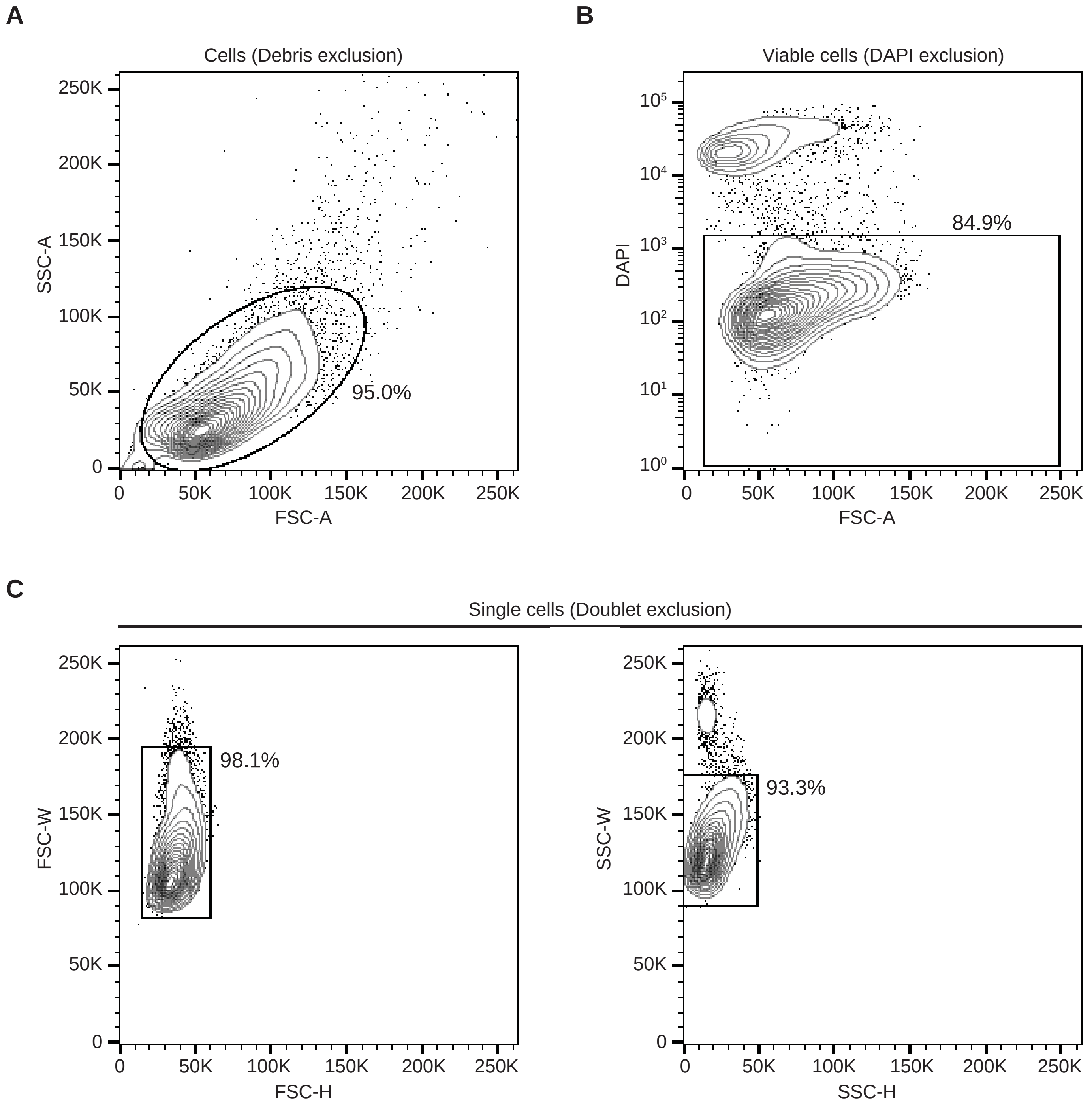

Figure S1. General gating strategy for all flow cytometric analyses (FACS) of human tracheal basal cells. First, cell were identified and gated away from debris by forward scatter (FSC) versus side scatter (SSC) (A). Viable cells were then gated by DAPI exclusion (B). Finally, single cells were gated by using FSC and SSC height-vs.-width plots to exclude doublets (C). Data are from α–CD44 staining shown in Additional file 7: Figure S2.
