# Supplementary material for: Cell surface marker profiling of human tracheal basal cells reveals distinct subpopulations, identifies MST1/MSP as a mitogenic signal, and identifies new biomarkers for lung squamous cell carcinomas
Source: Respir Res. 2014 Dec 31;15(1):160. doi: 10.1186/s12931-014-0160-8 (PMC4343068; doi:10.1186/s12931-014-0160-8)
Supplement: Additional file 7: Figure S2. — Pronase digestion reduces CD44 expression in tracheal basal cells. This figure presents FACS and immunostaining data that support pronase digestion cleaving epitopes on CD44. [file 12931_2014_160_MOESM7_ESM.pdf]

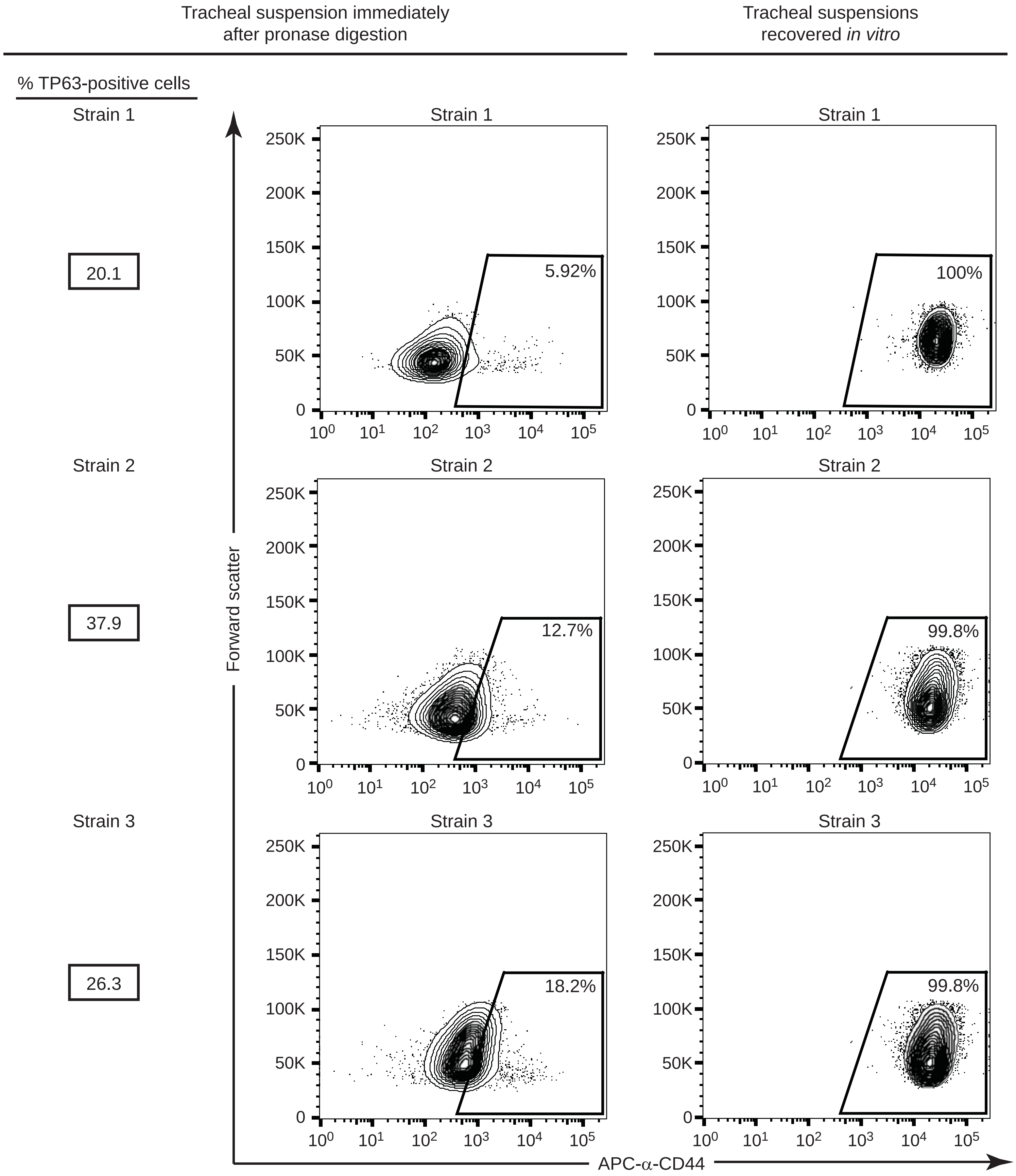

Figure S2. Pronase digestion reduces CD44 expression in tracheal basal cells. Cell suspensions freshly prepared from pronase digested tracheas were either cytospun and stained with anti-TP63 or stained with anti-CD44 antibodies and analyzed by FACS. 250-500 cells were counted per tracheal strain of basal cell. The fluorescence intensity of α-CD44 staining of non pronase-digested basal cells was determined by using recovered Passage 1 tracheal basal cells.
