# Supplementary material for: Cell surface marker profiling of human tracheal basal cells reveals distinct subpopulations, identifies MST1/MSP as a mitogenic signal, and identifies new biomarkers for lung squamous cell carcinomas
Source: Respir Res. 2014 Dec 31;15(1):160. doi: 10.1186/s12931-014-0160-8 (PMC4343068; doi:10.1186/s12931-014-0160-8)
Supplement: Additional file 8: Figure S3. — Primary human tracheal epithelial cell cultures consist entirely of CD44+ CD45- cells. This figure presents FACS data that indicate primary tracheal cell cultures are entirely comprised of CD44-positive and CD45-negative cells. [file 12931_2014_160_MOESM8_ESM.pdf]

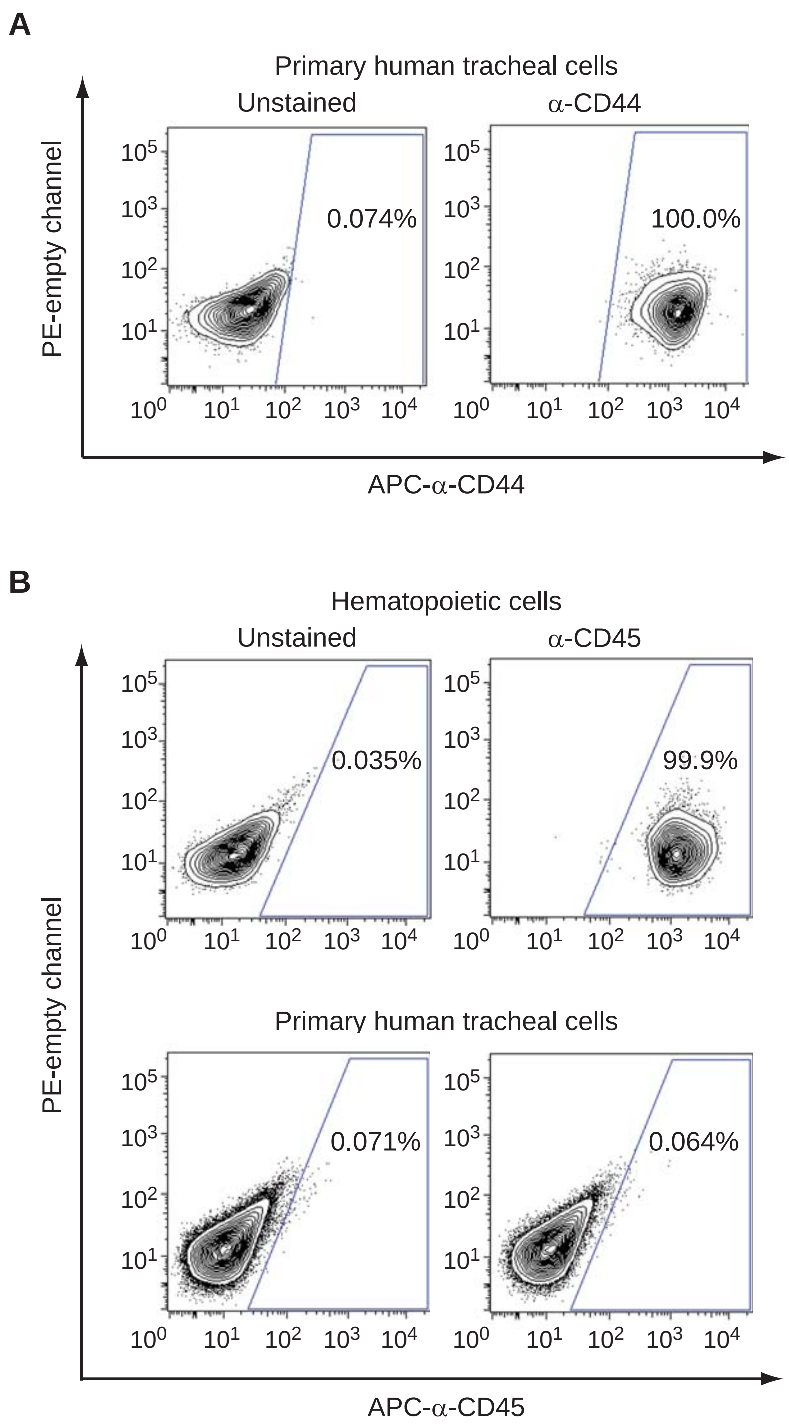

Figure S3. Primary human tracheal epithelial cell cultures consist entirely of CD44+ CD45- cells. Primary human tracheal cells were stained with α-CD44 (A) or α-CD45 (B) antibodies and analyzed by FACS. 8227 cells, an acute myeloid leukemia-derived hematopoietic cell line, were used as a positive control for α-CD45 staining.
