# Supplementary material for: Cell surface marker profiling of human tracheal basal cells reveals distinct subpopulations, identifies MST1/MSP as a mitogenic signal, and identifies new biomarkers for lung squamous cell carcinomas
Source: Respir Res. 2014 Dec 31;15(1):160. doi: 10.1186/s12931-014-0160-8 (PMC4343068; doi:10.1186/s12931-014-0160-8)
Supplement: Additional file 10: Figure S4. — AlamarBlue readings are directly proportional to tracheal basal cell number. This figure presents a standard curve that demonstrates a linear relationship between alamarBlue readings and tracheal basal cell number. [file 12931_2014_160_MOESM10_ESM.pdf]

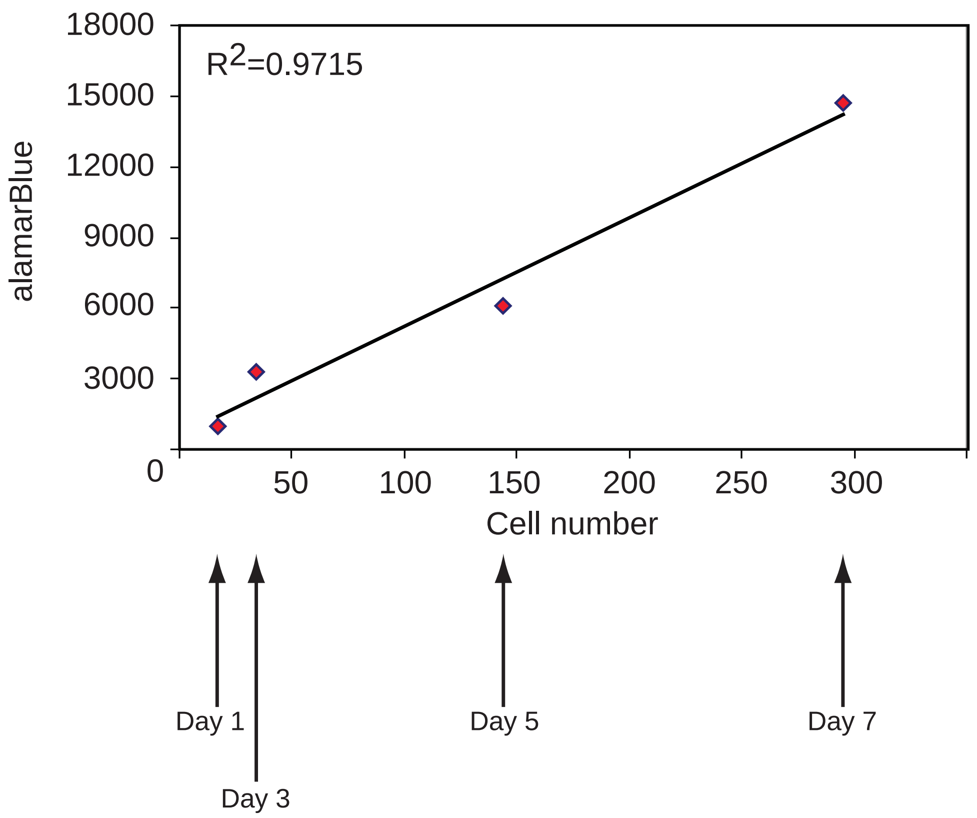


**Figure S4.** AlamarBlue readings are directly proportional to tracheal basal cell number. Human tracheal basal cells were grown *in vitro* in complete LHC-9 medium. Duplicate measurements of alamarBlue and cell number were taken at days 1, 3, 5, and 7 with the means shown.
